# Effect of mandibular advancement splint therapy on cardiac autonomic function in obstructive sleep apnoea

**DOI:** 10.1007/s11325-023-02924-y

**Published:** 2023-09-28

**Authors:** Seren Ucak, Hasthi U. Dissanayake, Kate Sutherland, Yu Sun Bin, Philip de Chazal, Peter A. Cistulli

**Affiliations:** 1 Charles Perkins Centre and Northern Clinical School, Faculty of Medicine and Health, University of Sydney, Sydney, Australia; 2 Department of Respiratory and Sleep Medicine, Royal North Shore Hospital, Sydney, Australia; 3 School of Biomedical Engineering, Faculty of Engineering, University of Sydney, Sydney, Australia

**Keywords:** Obstructive sleep apnoea, Mandibular advancement splint, Heart rate variability, Autonomic function, Cardiovascular disease

## Abstract

**Purpose:**

This study aimed to evaluate the effect of mandibular advancement splint (MAS) therapy on cardiac autonomic function in patients with obstructive sleep apnoea (OSA) using heart rate variability (HRV) analysis.

**Methods:**

Electrocardiograms (ECG) derived from polysomnograms (PSG) of three prospective studies were used to study HRV of patients with OSA before and after MAS treatment. HRV parameters were averaged across the entire ECG signal during N2 sleep using 2-min epochs shifted by 30 s. Paired *t*-tests were used to compare PSG and HRV measures before and after treatment, and the percent change in HRV measures was regressed on the percent change in apnoea-hypopnea index (AHI).

**Results:**

In 101 patients with OSA, 72% were Caucasian, 54% men, the mean age was 56 ± 11 years, BMI 29.8 ± 5.3 kg/m^2^, and treatment duration was 4.0 ± 3.2 months. After MAS therapy, there was a significant reduction in OSA severity (AHI, − 18 ± 16 events per hour, *p* < 0.001) and trends towards increased low-frequency to high-frequency ratio, low-frequency power, and reduced high-frequency power (LF:HF, − 0.4 ± 1.5, *p* = 0.01; LF, − 3 ± 16 nu, *p* = 0.02, HF, 3.5 ± 13.7 nu, *p* = 0.01). Change in NN intervals correlated with the change in AHI (*β*(SE) =  − 2.21 (0.01), *t* =  − 2.85, *p* = 0.005). No significant changes were observed in the time-domain HRV markers with MAS treatment.

**Conclusion:**

The study findings suggest that successful MAS treatment correlates with changes in HRV, specifically the lengthening of NN intervals, a marker for improved cardiac autonomic adaptability.

**Supplementary Information:**

The online version contains supplementary material available at 10.1007/s11325-023-02924-y.

## Introduction

Obstructive sleep apnoea (OSA) is a sleep-related breathing disorder characterized by the repeated collapse of the upper airway during sleep, leading to intermittent hypoxia, intrathoracic pressure swings, and fragmented sleep [1, 2]. OSA increases the risk of cardiovascular diseases, such as hypertension and coronary artery disease, partly due to changes in the autonomic nervous system, particularly upregulated sympathetic nerve activity [3].

Mandibular advancement splint (MAS) is an effective treatment for OSA and works by increasing upper airway patency and reducing collapsibility [4]. Currently, MAS is the recommended therapy for mild-moderate OSA and severe OSA when continuous positive airway pressure (CPAP) treatment is not tolerated [5]. Evidence suggests that health outcomes, at least in the short term, are comparable between MAS and CPAP therapy [6]. Randomized controlled trials of MAS treatment have demonstrated improved 24-h mean arterial blood pressure, which is more pronounced in hypertensive patients and those with resistant hypertension [7, 8]. However, the effects of MAS therapy on other intermediary cardiovascular markers, such as cardiac autonomic function, remain poorly understood [9], highlighting the need for further research on its potential to reduce cardiovascular risk.

Heart rate variability (HRV) analysis can be used non-invasively to evaluate beat-to-beat changes in cardiac autonomic control, which is particularly valuable for evaluating the relative activity of sympathetic and parasympathetic cardiac modulation [10]. OSA is associated with an HRV profile indicative of sympathetic predominance, which is a hallmark of hypertension, a major risk factor for cardiovascular disease [11]. Therefore, we aimed to use HRV to assess cardiac autonomic function following MAS therapy. We hypothesized that cardiac autonomic function would improve following successful MAS treatment i.e. that there would be a reduction in sympathetic and an increase in parasympathetic HRV markers.

## Methods

### Participants

**Fig. 1:**
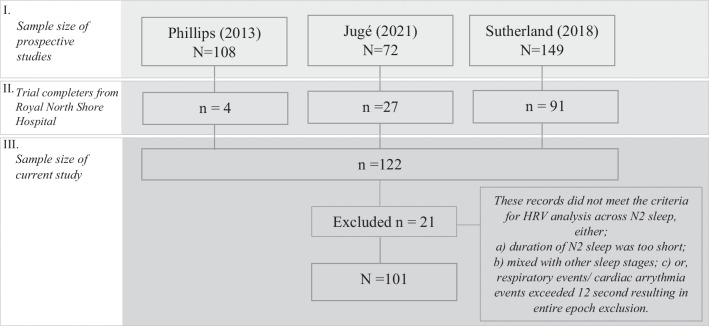
Study flow

This study utilized data from three prospective MAS therapy research studies conducted at the Sleep Investigation Laboratory, Royal North Shore Hospital. Adult participants (age ≥ 18 years) diagnosed with OSA (AHI ≥ 10/h) and treated with MAS were included in the current analysis (Fig. 1). These studies collected baseline polysomnography (PSG) upon initial visit to the clinic, and this was followed by a treatment PSG with MAS after at least one month of MAS therapy [4, 12, 13]. Sutherland et al. [12] used this data to derive multimodal phenotypes to predict treatment response in patients using MAS. Phillips [4] performed a randomized controlled trial (RCT) comparing health outcomes of MAS and CPAP treatment. Jugé [13] used imaging techniques to characterize whether inspiratory tongue movement is associated with MAS treatment outcomes. Study characteristics and outcomes were compared across these three datasets and are presented in the Supplementary Material (Supplementary Tables 1–2). In all studies, dental eligibility was assessed by a dentist, and exclusion criteria were limited to contraindications to MAS therapy i.e. periodontal disease, temporomandibular joint diseases, active upper airway infection, and insufficient teeth.

We used the same MAS design for all studies — a titratable, custom-fitted two-piece device (SomnoMed Ltd., based in Sydney, Australia). Participants incrementally adjusted the MAS to the point of maximum comfortable mandibular advancement, as a means of self-titration and titration degree, with the titration degree confirmed by a dental specialist to ensure the correct adjustment. All studies were approved by Institutional Human Research Ethics Committees, and informed consent was obtained from all participants before the trials commenced.

For the present study, the participants included patients of varying response to treatment. Study characteristics and outcomes for different response groups were compared and presented in the Supplementary Material (Supplementary Table 3–4).

### Polysomnography

During full night PSG, standard channels were utilized to record physiological parameters, including electrooculography (EOG), chin electromyography (EMG), nasal airflow pressure via nasal cannula, thoracic and abdominal respiratory effort, finger pulse oximetry to measure oxygen saturation (SpO2%), body position, and leg electromyography. Trained sleep scientists scored each recording according to the guidelines set by the American Academy of Sleep Medicine [14].

#### Holter monitor processing

As part of in-laboratory PSG, electrocardiogram (ECG) data was collected using three electrodes placed in the standard lead II configuration. The ECG signals were extracted from the polysomnograms and analysed using commercially available Holter software for QRS detection, ectopic beat detection, and labelling (Sentinel Holter Data Management System v11.5.1, Spacelabs Healthcare, Issaquah, WA, USA). QRS detection was performed with a resolution of 1 ms.

### Heart rate variability

HRV analysis was performed on ECG recordings in accordance with standard guidelines [10] using HRV algorithms implemented in signal processing software (MATLAB 2017, version 9.2.0.538062 (R2017a), Mathworks, Natick, Massachusetts). HRV parameters were calculated using 2-min epochs, shifted by 30 s, across the entire ECG signal, and then averaged across segments of N2 sleep (stage 2 non-rapid eye movement). N2 sleep is the preferred stage for nocturnal HRV analysis due to its relative stability, as it is primarily controlled by the parasympathetic nervous system and has fewer arousals that may impact the sensitivity of HRV measures [15]. Epochs with mixed sleep stages and ECG sections associated with respiratory events or cardiac arrhythmia (including atrial and ventricular ectopic beats) were excluded from HRV analysis. If the total exclusion period exceeded 12 s (10% of epoch length), the entire epoch was excluded [16].

#### Time — domain measures

The time domain HRV metrics employed in the analysis were (1) Average NN interval (2) SDNN, standard deviation of NN intervals, utilized as a measure of global HRV, (3) RMSSD, which is the square root of the mean squared differences of successive NN intervals, and (4) pNN50, the percentage of absolute differences in successive NN values greater than 50 ms (Table 1). RMSSD and pNN50 are short-term measures that are strongly associated with high-frequency oscillations and reflect parasympathetic modulation of the heart [10].

**Table 1:** Description of time and frequency domain HRV measures. Adapted from Malik et al. 1996 [10]

	Description	Physiological interpretation
Time domain measures		
Average NN interval, ms	Average time between consecutive R-peaks	Primarily parasympathetic cardiac modulation
SDNN, ms	Standard deviation of normal to normal intervals	Global HRV measure
RMSSD, ms	Root mean square of successive RR interval differences	Primarily parasympathetic cardiac modulation
pNN50, %	Percentage of successive RR interval that differ by more than 50 ms	Primarily parasympathetic cardiac modulation
Frequency domain measures		
TP, ms2	The absolute power of the frequency spectrum, excluding the very low frequency band (> 0.004 Hz)	Global HRV measure
LF, ms2	Absolute power of the low-frequency band (0.04–0.15 Hz)	Sympathetic with a parasympathetic component
HF, ms2	Absolute power of the high-frequency band (0.15–0.4 Hz)	Primarily parasympathetic cardiac modulation
LF:HF, nu	Ratio of LF-to-HF power	Sympathovagal balance
LF, nu	Relative power of the low-frequency band (0.04–0.15 Hz) in normal units	Primarily sympathetic cardiac modulation
HF, nu	Relative power of the high-frequency band (0.15–0.4 Hz) in normal units	Primarily parasympathetic cardiac modulation

#### Frequency — domain measures

Frequency-domain HRV parameters were calculated using the Lomb periodogram on the preprocessed data. Total power (TP, ms^2^) was defined as the power between 0.04 and 0.4 Hz and is associated with global HRV changes. Absolute power within the low frequency band (0.04–0.15 Hz; LF, ms^2^) reflects sympathetic cardiac modulation with a vagal component (Table 1), while absolute power within the high frequency band reflects vagal cardiac autonomic control (0.15–0.40 Hz; HF, ms^2^. Normalized values LFnu and HFnu are associated with sympathetic and parasympathetic activity, respectively [10]. The ratio between these indices (LF: HF) represent sympathovagal balance [10].

### Statistical analysis

Mean and standard deviation (SD) were presented for continuous variables. Paired *t*-tests were utilized to compare polysomnographic characteristics and HRV markers before and after MAS treatment. Normality was evaluated using the Shapiro–Wilk test, and distribution plots were visually examined. Wilcoxon signed rank test was used for pairwise comparisons of nonparametric continuous data.

To investigate whether the responsiveness to MAS treatment influenced the changes in HRV, regression analyses were conducted for the change in HRV markers (∆HRV) (outcome variable) against treatment duration (predictor), and against treatment duration and change in AHI (∆AHI) (predictors). Treatment duration was calculated as the amount of time, in months, between the PSG collected at baseline and with treatment. The change ($\Delta$) was calculated using the following formula: $\Delta = T-B$ where $T$ referred to treatment outcomes and $B$ indicated baseline outcomes. The *p* value was adjusted for multiple comparisons using Bonferroni correction; therefore, significance is denoted as *p* values less than 0.005 (10 comparators). Subgroup analyses were also performed comparing clinical and HRV outcomes between (1) all three data sources, (2) treatment response groups, and (3) treatment duration groups (Supplementary Tables 1 – 5). All data analyses were performed using SPSS software (version 24; SPSS Inc., Chicago, IL).

## Results

### Participant characteristics

Table 2 shows the clinical characteristics of the 101 study participants. The sample had a relatively even distribution of men and women (54% men), who were mostly middle-aged (age, 56 ± 11 years), overweight (BMI, 29.8 ± 5.3 kg/m^2^) and identified themselves as Caucasian (72% Caucasian). The average duration of treatment was 4.0 ± 3.2 months.

**Table 2:** Clinical characteristics of the entire sample (*N* = 101). Continuous data are presented as mean (SD); categorical data are presented as count (percentage, %)

Characteristics	*N* = 101
Age, years	56 (11)
Sex, male %	54 (52)
BMI, kg/m^2^	29 (5)
Ethnicity, Caucasian %	72 (71)
Treatment time, months	4 (3)

### MAS treatment effect on polysomnographic indices

Table 3 shows the changes in polysomnographic parameters observed from baseline to treatment. The results show a significant reduction in AHI, AHI_NREM_, AHI_REM_, and ODI4% (mean difference ± SE; − 18 ± 17); − 19 ± 24; − 19 ± 21; − 13 ± 15 events/hour), and a significant increase in SpO_2_ nadir (5 ± 33%). No significant changes were observed in TST, NREM duration, REM duration, or sleep efficiency.

**Table 3:** Comparing polysomnographic indices. Paired *t*-tests were used to compare continuous polysomnographic indices presented as mean and standard deviation (SD). The Wilcoxon signed rank test was used for nonparametric data denoted ‘^a^’ and presented as median and interquartile range (IQR). Test statistic (*t*) and degrees of freedom (df) presented for parametric paired *t*-tests and the *H* statistic (*H*) was used for nonparametric analyses. *p* values adjusted for multiple comparisons using Bonferroni correction; significance is denoted as **p < 0.005

PSG parameters	Baseline	Treatment	Test statistic	*p*
	Mean (SD)/Median (IQR)	Mean (SD)/Median (IQR)		
AHI	27 (17)	10 (11)	− 8.74^a^	< .001**
AHI_NREM_	27 (20)	6 (12)	− 6.43^a^	< .001**
AHI_REM_	42 (41)	15 (30)	− 6.39^a^	< .001**
ODI 4%	13 (20)	7 (9)	− 3.89^a^	< .001**
TST, min	362 (550)	367 (82)	0.19 (100)	0.85
NREM, min	298 (38)	297 (64)	1.23 (100)	0.23
REM, min	63 (28)	67 (30)	− 1.78 (100)	0.09
Sleep efficiency, %	77 (18)^ŧ^	78 (17)^ŧ^	− 1.01^a^	0.31
SpO2 nadir, %	83 (5)	87 (6)	− 6.42 (100)	< .001**

### MAS treatment effect on HRV measures

Table 4 shows the changes in HRV markers before and after MAS treatment. The results show a trend towards increased LF:HF ratio and LFnu (0.4 ± 1.5, 3 ± 16nu), along with a reduction in HFnu (− 3.5 ± 13.7nu). No significant changes were observed in the time-domain HRV markers with MAS treatment.

**Table 4:** Comparing HRV markers. Paired *t*-tests were used to compare HRV measures before and with MAS treatment and presented as mean and standard deviation (SD). The Wilcoxon signed rank test was used for nonparametric data denoted ‘^a^’ and presented as median and interquartile range (IQR). Test statistic (*t*) and degrees of freedom (df) presented for parametric paired *t*-tests and the *H* statistic (*H*) was used for nonparametric analyses. *p* values adjusted for multiple comparisons using Bonferroni correction, significance is denoted as * *p* < 0.005

HRV markers	Baseline	Treatment		
	Mean (SD)/Median (IQR)	Mean (SD)/Median (IQR)	Test statistic	*p*
avgNN, ms	982 (173)	1017 (181)	− 1.64^a^	0.100
SDNN, ms	44 (27)	42 (31)	− 2.20^a^	0.028
RMSSD, ms	34 (24)	31 (24)	− 2.03^a^	0.042
pNN50, %	12 (24)	9 (22)	− 1.83^a^	0.068
TP, ms^2^	2237 (2651)	1991 (2951)	− 1.98^a^	0.048
LF, ms^2^	607 (998)	559 (1186)	− 1.80^a^	0.071
HF, ms^2^	494 (698)	355 (635)	− 2.65^a^	0.008
LF:HF	1.2 (1.3)	1.4 (1.4)	− 2.16^a^	0.030
LF_nu_	51 (17)	54 (18)	− 2.02 (100)	0.046
HF_nu_	42 (15)	39 (16)	2.56 (100)	0.012

**Fig. 2:**
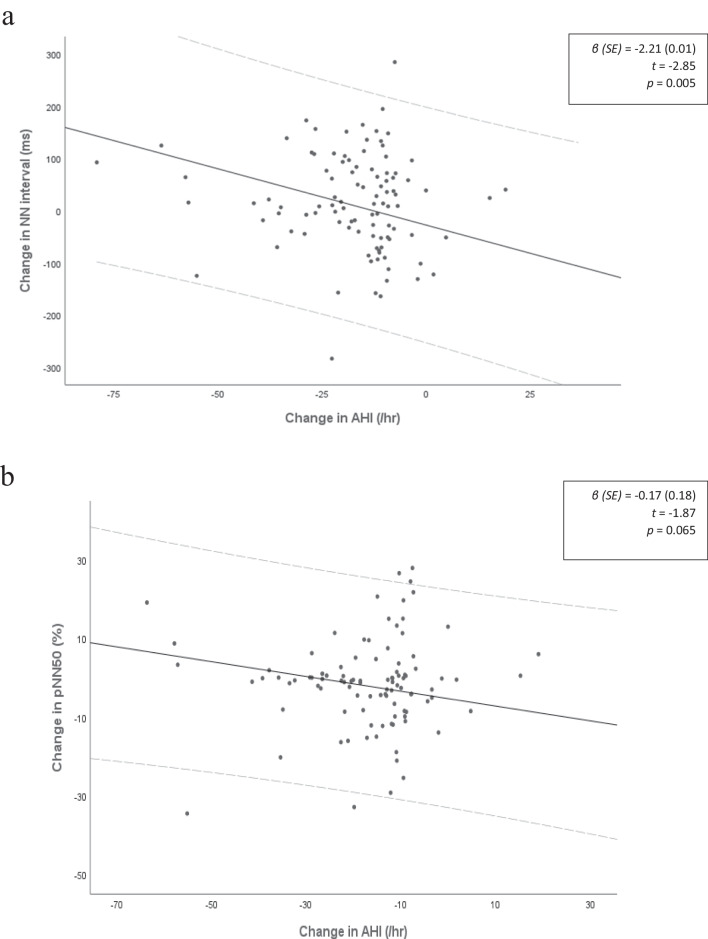
Scatter plot of change in HRV by change in AHI. The trend line is presented with 95% confidence intervals; significance is denoted by an * as *p* < 0.005. **a** Change in NN interval by change in AHI. **b** Change in pNN50 by change in AHI

Regression analyses comparing changes in HRV and AHI measures are presented in Table 5. The results indicate a correlation between change in average NN interval and change in AHI (*β*(SE) =  − 2.21 (0.01), *t* =  − 2.85, *p* = 0.005) (Fig. 2). Change in pNN50 and change in AHI also trend towards an association (*β*(SE) =  − 0.17 (0.18), *t* =  − 1.87, *p* = 0.065) (Fig. 2). Change in AHI following MAS treatment was not significantly correlated with changes in frequency domain HRV measures. In addition, treatment duration had no association with HRV measures.

**Table 5:** Change in HRV markers regressed against change in AHI. *p* values adjusted for multiple comparisons using Bonferroni correction; significance is denoted as * *p* < 0.005

Change in HRV markers	Model 1^a^			Model 2^b^		
	β (SE)	*t*	*p*	*β* (SE)	*t*	*p*
ΔavgNN, ms	− 0.07 (4.84)	− 2.84	0.989	− 2.21 (0.78)	− 2.85	0.005
ΔSDNN, ms	− 0.01 (0.28)	− 0.32	0.752	− 0.02 (0.03)	− 0.66	0.510
ΔRMSSD, ms	0.21 (3.56)	0.06	0.955	− 0.42 (0.57)	− 0.72	0.471
ΔpNN50, %	0.21 (0.58)	0.35	0.720	− 0.17 (0.09)	− 1.87	0.065
ΔTP, ms^2^	− 226.96 (928.02)	− 0.24	0.807	− 0.01 (0.01)	− 0.42	0.671
ΔLF, ms^2^	− 85.73 (243.14)	− 0.04	0.725	− 0.06 (0.01)	− 0.59	0.550
ΔHF, ms^2^	− 122.29 (558.97)	− 0.21	0.827	− 0.05 (0.01)	− 0.45	0.648
ΔLF:HF	0.09 (0.07)	1.43	0.156	− 0.22 (1.01)	− 0.22	0.822
ΔLF_nu_	0.62 (0.68)	− 0.63	0.525	− 0.03 (0.10)	− 0.36	0.719
ΔHF_nu_	− 0.33 (0.60)	− 0.55	0.580	− 0.09 (0.11)	− 0.84	0.401

^a^Model 1. Change in HRV marker as the outcome variable and treatment duration as a predictor

^b^Model 2. Change in HRV marker as the outcome variable and treatment duration and change in AHI as a predictor

Supplementary Table 5 (refer to Supplementary Material) compares changes in HRV markers across patients grouped by treatment duration. There were no significant differences between change in HRV markers across the three treatment time groups. However, there was a trend towards higher change in average NN interval in the longer term, 9–12-month treatment group. Although this group also had the lowest sample size with only 6 participants.

## Discussion

This study investigated the effect of MAS treatment on cardiac autonomic function in patients with OSA. Our hypothesis was that successful MAS treatment would result in increased parasympathetic and reduced sympathetic cardiac autonomic modulation. The findings revealed a novel correlation between successful MAS therapy and lengthened NN intervals, a marker of improved cardiac autonomic adaptability [17]. Specifically, each unit of reduction in AHI was associated with a 2.21 unit increase in NN interval length, and a similar trend towards higher pNN50 with improved AHI. The lengthened NN interval observed resulted from increased cardiac vagal modulation, which reflected improved cardiac autonomic adaptability and may have implications for reducing cardiovascular risk [17].

Of the five similar studies undertaken [18], only one study has reported nocturnal vagal predominance after MAS use [19]. The authors showed in their retrospective study that after 3 months of MAS use (*N* = 58), patients demonstrated improved cardiac autonomic adaptability through increases in average NN, normalized HF power, and SDNN and reduced normalized LF power [19]. In addition, other studies have reported an increase in daytime normalized HF power after short-term MAS use, albeit using small sample sizes (*N* = 10) and only during a controlled breathing protocol, rather than normal breathing [19, 20]. In the other studies investigating changes to HRV markers after MAS, no changes to sympathetic or vagal markers were found [21, 22]. In their randomized crossover trial, Dal-Fabbro and colleagues [21] showed that compared with placebo oral appliance, global HRV marker, TP, was significantly lower, with no differences found between MAS and CPAP treatment periods. Collectively, these findings suggest that MAS use may result in improved cardiac autonomic adaptability, though different studies have yielded different conclusions.

Clinically, MAS use is hindered by its varying degrees of efficacy whereby around one-third of patients completely resolve OSA with MAS treatment, one-third will have a demonstrated halving of their OSA severity, and one-third will demonstrate minimal effect [23]. Therefore, patient responsiveness to MAS therapy must be considered when evaluating treatment effect on cardiac autonomic function. In our study, improvement in vagally mediated HRV markers was only observed after considering responsiveness to MAS treatment (i.e., those with a greater change in AHI). We found a trend towards increased pNN50 with improved AHI. Similar subgroup analyses of treatment responders (*n* = 34, defined as, > 50% reduction in AHI and AHI < 20/h after treatment) identified significant increases in average NN and normalized HF power, accompanied by reductions in TP, VLF, LF, LF:HF ratio, and normalized LF power after MAS treatment [19]. In contrast, no significant changes in cardiac autonomic function were observed in the non-responder group (*n* = 24), defined as those with a < 50% reduction in AHI and AHI > 20/h following treatment [19]. Taken together, these two investigations suggest responders to MAS treatment experience a marked shift towards parasympathetic predominance in sympathovagal balance, characteristic of improved cardiac autonomic regulation. However, the lack of uniformity in results across all MAS treatment trials suggests that responsiveness to MAS therapy is only one factor contributing to improved cardiovascular health.

Despite an inextricable link between OSA and cardiovascular risk, the largest randomized study to date with a 3.7-year follow-up period investigating OSA treatment failed to demonstrate any improvement in hard cardiovascular endpoints [24]. A significant limitation in this trial was poor treatment adherence to CPAP, which was only 3.3 h/night in a population with cardiovascular disease [24]. While MAS therapy is generally less efficacious than CPAP, its higher treatment adherence makes both treatment modalities equally effective [23]. The main set-back to ours and other MAS treatment trials has been a lack of access to technologies to objectively record daily adherence. Therefore, future MAS trials should consider the effects of treatment responsiveness and objective daily adherence when evaluating cardiovascular risk.

HRV is a useful tool to monitor the cardiac autonomic benefits of OSA treatment, yet there is substantial heterogeneity among treatment trials [18]. Differences in HRV indices used and HRV sensitivity to external factors affecting HRV measurements are observed. Not only do methodologies vary between whether or not HRV is measured during daytime, nighttime, or over 24 h [18]. Some studies used single 5-min segments to represent changes that occur over whole sleep stages [21], while others used a minimum number of beats per tachogram [25], or limited their nocturnal analyses to specific times irrespective of sleep stages [19]. This is problematic as time domain analysis of HRV is highly influenced by the condition and duration of the recording [10]. Routine recording of ECG during polysomnography makes assessment of HRV during sleep a widely used and easily accessible method for measuring cardiac autonomic function. However, disordered respiration experienced by patients with OSA reduces the sensitivity of HRV as a tool for evaluating the effectiveness of OSA therapy on global measures of HRV [10]. We addressed this concern by excluding ECG segments with respiratory events, ectopic beats, and other cardiac arrhythmias. In order to increase sensitivity of our HRV measure, we also adopted a methodology of averaging HRV markers across the entire PSG-derived ECG using a shifting window of 2 min, and generating HRV measurements for the N2 sleep stage, as is common practice [15]. However, our study could have benefitted from longer duration of data collection. Using 24-h Holter monitoring to assess the SDNN as a marker of global HRV is preferable for HRV assessment, as it can better account for metabolic and circadian variability, which are strongly associated with cardiovascular risk [10].

In some patients, MAS treatment can alter nocturnal cardiac autonomic activity. Multiple studies have shown that MAS treatment may enhance vagally mediated markers, thereby improving cardiac autonomic adaptability. However, research in the field of MAS is hampered by various challenges. As previously highlighted, the lack of objective daily usage data is a major limitation to this and other similar studies. While some studies have provided preliminary data on average usage using objective adherence monitors for up to 1 year of treatment, these studies were small and did not evaluate cardiovascular outcomes. In contrast, our study had a relatively large sample size and captured a more comprehensive and accurate measure for cardiac autonomic function by averaging HRV markers over the entire ECG. However, our study did use data sets from three separate treatment trials and adherence was not measured uniformly across these studies. Therefore, we were unable to provide objective or subjective measures of adherence.

## Conclusion

The current study investigated the effect of MAS treatment on cardiac autonomic function in patients with OSA. The findings suggested that successful treatment with MAS correlated with changes to HRV, specifically the increase in pNN50 and the lengthening of NN intervals, a marker for improved cardiac autonomic adaptability [10, 17]. However, change in AHI following MAS treatment was not significantly correlated with changes in frequency domain HRV measures. 

### Supplementary Information

Below is the link to the electronic supplementary material.

Supplementary file1 (DOCX 33 KB)

Supplementary file2 (DOCX 14 KB)

Supplementary file3 (DOCX 15 KB)

Supplementary file4 (DOCX 14 KB)

Supplementary file5 (DOCX 15 KB)

Supplementary file6 (DOCX 15 KB)

## Data Availability

The datasets made available for this study and/or generated analysis during the current study are available from the corresponding author on reasonable request.
