# Supplementary material for: Effect of mandibular advancement splint therapy on cardiac autonomic function in obstructive sleep apnoea
Source: Sleep Breath. 2023 Sep 28;28(1):349–57. doi: 10.1007/s11325-023-02924-y (PMC10955011; doi:10.1007/s11325-023-02924-y)
Supplement: Supplementary file 1 — Supplementary file1 (DOCX 33 KB) [file 11325_2023_2924_MOESM1_ESM.docx]

**SUPPLEMENTARY MATERIAL**

**Comparison of data set sources**

Additional analyses were conducted comparing clinical (Supplementary Table 1) and HRV markers (Supplementary Table 2) between the results from the three treatment trials used for this study. Clinical characteristics such as age, BMI, treatment time, sex and ethnicity were included in the analysis. The change in each HRV marker was compared across the three studies.

Treatment time was significantly longer in Jugé’ study compared with Sutherland and Philips. Participants were significantly older in Sutherlands’ study compared with Jugé. There were no significant differences in other clinical characteristics. There were no significant differences in changes to HRV markers (∆HRV). It is important to note the differences in sample sizes between the three groups, in particular the small sample of Phillips’ study which might not be sufficiently powered to reflect any differences.

**Subgroup analyses of response groups**

The 101-participant analysed for this study had varying degrees of responsiveness to MAS treatment. Additional subgroup analyses were undertaken to compare differences in clinical characteristics between the three response groups (Supplementary Table 3). In addition, HRV markers were assessed before and after treatment for each subgroup and tabulated (Supplementary Table 4). Response to treatment was determined by degree of improvement in AHI according to previously reported rigorous definition by Sutherland (2011). Definitions are as follows:

1. Complete responders (n = 36) - patients with > 50% improvement in AHI as well as a posttreatment AHI < 5/h.
2. Partial responders (n =38) – patients with > 50% AHI reduction but still had residual OSA (AHI > 5/h).
3. Non- Responders (n= 27) – patients with < 50% AHI reduction.

There were no significant differences in clinical characteristics between response groups, however the BMI for the partial response group was slightly higher than that of the complete or non-responder groups (Supplementary Table 3). Likewise, there were no significant difference between change in HRV markers between the response groups (Supplementary Table 4).

**Subgroup analyses of treatment time**

Our results suggest that when the regression model takes (∆HRV) as the outcome variable and treatment time and ∆AHI as the predictor, ∆NN changes significantly. The treatment duration in months, seems to have some effect on HRV markers , therefore we performed additional subgroups analyses on three groups based on their overall treatment duration in months. The minimum treatment time for this study is one month, the maximum is 12 months. The analyses were performed on the following groups:(1) 1-4 months treatment; (2) 5-8 months treatment; (3) 9-12 months treatment (Supplementary Table 5).

There were no significant differences between change in HRV markers across the three treatment time groups. However, there was a trend towards higher change in average NN interval in the longer term, 9–12-month treatment group. Although this group also had the lowest sample size with only 6 participants.

|  | **Sutherland 2018 n = 73** | **Jugé 2021 n = 25** | **Phillips 2013 n = 3** | **Test Statistic** | **p** |
| --- | --- | --- | --- | --- | --- |
| **Age, years** | 58 (14) | 50 (18) | 60 (10) | 5.3 (2, 98) | 0.007 ^c^ |
| **Sex, male %** | 40 (55) | 13 (52) | 1(33) | 0.6 (2) | 0.753 |
| **BMI, kg/m²** | 29 (7) | 27 (7) | 30 (8) | 0.7 (2, 98) | 0.482 |
| **Ethnicity, Caucasian %** | 56 (78) | 16 (64) | 3 (100) | 3.8 (2) | 0.151 |
| **Treatment time, months** | 3 (3) | 4 (3) | 1 (0) | 6.6 (2, 98) | <0.001*^b,c^ |

**Supplementary Table 1.** The table compares clinical characteristics across the three studies. Parametric variables were compared using one-way ANOVA and reported as mean (standard deviation, SD) and F statistic (df; degrees of freedom between groups, within groups). Results for Phillips 2013 were reported as median (minimum - maximum). Categorical variables were compared across the three groups using Chi-Square tests and reported as count (percentage, %), and Chi-square test static, χ^2^ (df). Significance denoted, * p<0.005.  ^b A significant difference between Jugé, 2021 and Phillips, 2013
c A significant difference between Jugé, 2021 and Sutherland, 2018
d A significant difference between Sutherland 2018 and Phillips, 2013^

| **Change in HRV** | **Sutherland 2018 n = 73** | **Jugé 2021 n = 25** | **Phillips 2013 n = 3** | **Test Statistic** | **p** |
| --- | --- | --- | --- | --- | --- |
| **avgNN _ms_** | 9 (131) | 21 (121) | 71 (25-164) | 2.2  ^a^ | 0.325 |
| **SDNN _ms_** | -8 (13) | -3 (13) | 18 (-1–268) | 3.8  ^a^ | 0.146 |
| **RMSSD _ms_** | -4 (14) | -1 (14) | 9 (2-406) | 4.5  ^a^ | 0.106 |
| **pNN50 ^%^** | -1 (11) | -1 (9) | 5 (1-22) | 3.4  ^a^ | 0.181 |
| **TP _ms_^2^** | -258 (1502) | -297 (1151) | 1585 (-288-142219) | 2.9  ^a^ | 0.234 |
| **LF _ms_^2^** | -51 (458) | -56 (233) | 599 (-39-36668) | 3.4  ^a^ | 0.179 |
| **HF _ms_^2^** | -90 (432) | -43 (357) | 150 (38 -86273) | 4.3  ^a^ | 0.118 |
| **LF: HF** | -1 (1) | -1 (1) | -1 (-1-1) | 1.8  ^a^ | 0.399 |
| **LF_nu_** | 4 (16) | 3 (11) | -6 (23) | 0.6 (2, 98) | 0.545 |
| **HF_nu_** | -5 (15) | -1 (7) | 10 (22) | 2.1 (2, 98) | 0.124 |

**Supplementary Table 2.** The table compares change in HRV markers across the three studies. Nonparametric variables were compared using Kruskal-Wallis Test, denoted ^‘a’^, and reported as median (interquartile range, IQR ) with the H test statistic. Parametric variables were compared using one-way ANOVA and reported as mean (standard deviation, SD) and F statistic (df; degrees of freedom between groups, within groups). Results for Phillips 2013 were reported as median (minimum - maximum). Significance denoted, * p<0.005

|  | **Complete Responder**  **N= 36** | **Partial Responder**  **N = 38** | **Non-Responder**  **N= 27** | **Test Statistic** | **p** |
| --- | --- | --- | --- | --- | --- |
| **Age, years** | 53 (12) | 56 (10) | 57 (13) | 0.8 (2, 98) | 0.461 |
| **Sex, male %** | 19 (53) | 18 (47) | 17 (62) | 1.1 (2) | 0.568 |
| **BMI, kg/m²** | 27 (4) | 31 (6) | 30 (5) | 4.7 (2, 98) | 0.011 |
| **Ethnicity, Caucasian %** | 26 (72) | 28 (73) | 18 (67) | 11.4 (2) | 0.524 |
| **Treatment time, months** | 3 (2) | 4 (2) | 4 (2) | 1.1 (2, 98) | 0.450 |

**Supplementary Table 3.** The table compares clinical characteristics across the three response groups. Parametric variables were compared using one-way ANOVA and reported as mean (standard deviation, SD) and F statistic (df; degrees of freedom between groups, within groups). Categorical variables were compared across the three groups using Chi-Square tests and reported as count (percentage, %), and Chi-square test static, χ^2^ (df). Significance denoted, * p<0.005

| **Change in HRV** | **Complete Responder**  **n= 36** | **Partial Responder**  **n = 38** | **Non-Responder**  **n= 27** | **Test Statistic** | **p** |
| --- | --- | --- | --- | --- | --- |
| **avgNN _ms_** | 45 (173) | 13 (114) | 5 (120) | 2.9  ^a^ | 0.234 |
| **SDNN _ms_** | -3 (18) | -4 (15) | -7 (10) | 1.2  ^a^ | 0.532 |
| **RMSSD _ms_** | -2 (25) | -2 (11) | -3 (12) | 0.3  ^a^ | 0.831 |
| **pNN50 ^%^** | -1 (16) | -1 (8) | -3 (11) | 1.4  ^a^ | 0.492 |
| **TP _ms_^2^** | -259 (2227) | -95 (1282) | -499 (982) | 1.2  ^a^ | 0.540 |
| **LF _ms_^2^** | -66 (421) | -3 (376) | -88 (334) | 2.0  ^a^ | 0.362 |
| **HF _ms_^2^** | -22 (1067) | -50 (316) | -137 (333) | 0.9  ^a^ | 0.909 |
| **LF: HF** | 1 (1) | 1 (1) | -1 (1) | 3.1  ^a^ | 0.210 |
| **LF_nu_** | 3 (15) | 4 (13) | 1 (15) | 0.6 (2, 98) | 0.540 |
| **HF_nu_** | -5 (14) | -5 (15) | -4 (12) | 0.5 (2, 98) | 0.612 |

**Supplementary Table 4.** The table compares change in HRV markers across the three response subgroups. Nonparametric variables were compared using Kruskal-Wallis Test, denoted ^‘a’^, and reported as median (interquartile range, IQR ) with the H test statistic. Parametric variables were compared using one-way ANOVA and reported as mean (standard deviation, SD) and F statistic (df; degrees of freedom between groups, within groups). Significance denoted, * p<0.005
Complete responders (n = 36), defined as patients with > 50% improvement in AHI as well as a posttreatment AHI < 5/h; Partial responders (n =38), defined as patients with > 50% AHI reduction but still had residual OSA (AHI > 5/h), Non- Responders (n= 27), defined as patients with < 50% AHI reduction.

| **Change in HRV** | **1-4 months**  **n= 74** | **5-8 months n = 21** | **9-12 months n= 6** | **Test Statistic** | **p** |
| --- | --- | --- | --- | --- | --- |
| **avgNN _ms_** | 14 (128) | 5 (125) | 90 (131) | 5.1  ^a^ | 0.078 |
| **SDNN _ms_** | -4 (13) | -2 (15) | 5 (14) | 2.7  ^a^ | 0.257 |
| **RMSSD _ms_** | -3 (15) | -2 (18) | -2 (22) | 1.6  ^a^ | 0.449 |
| **pNN50 ^%^** | -1 (11) | -1 (14) | -1 (24) | 1.9  ^a^ | 0.392 |
| **TP _ms_^2^** | -309 (1462) | -120 (1555) | 403 (1330) | 3.2  ^a^ | 0.200 |
| **LF _ms_^2^** | -68 (360) | -51 (343) | 127 (385) | 2.9  ^a^ | 0.235 |
| **HF _ms_^2^** | -92 (400) | -46 (530) | -32 (637) | 1.8  ^a^ | 0.399 |
| **LF: HF** | 1 (1) | 1 (1) | 1 (3) | 0.2  ^a^ | 0.917 |
| **LF_nu_** | 3 (16) | 3 (14) | 11 (18) | 0.7 (2, 98) | 0.488 |
| **HF_nu_** | -3 (14) | -4 (16) | -3 (14) | 0.1 (2, 98) | 0.866 |

**Supplementary Table 5.** The table compares change in HRV markers across the three treatment time groups. Nonparametric variables were compared using Kruskal-Wallis Test, denoted ^‘a’^, and reported as median (interquartile range, IQR ) with the H test statistic. Parametric variables were compared using one-way ANOVA and reported as mean (standard deviation, SD) and F statistic (df; degrees of freedom between groups, within groups). Significance denoted, * p<0.005
