# Supplementary material for: Effect of mandibular advancement splint therapy on cardiac autonomic function in obstructive sleep apnoea
Source: Sleep Breath. 2023 Sep 28;28(1):349–57. doi: 10.1007/s11325-023-02924-y (PMC10955011; doi:10.1007/s11325-023-02924-y)
Supplement: Supplementary file 2 — Supplementary file2 (DOCX 14 KB) [file 11325_2023_2924_MOESM2_ESM.docx]

|  | **Sutherland 2018 n = 73** | **Jugé 2021 n = 25** | **Phillips 2013 n = 3** | **Test Statistic** | **p** |
| --- | --- | --- | --- | --- | --- |
| **Age, years** | 58 (14) | 50 (18) | 60 (10) | 5.3 (2, 98) | 0.007 ^c^ |
| **Sex, male %** | 40 (55) | 13 (52) | 1(33) | 0.6 (2) | 0.753 |
| **BMI, kg/m²** | 29 (7) | 27 (7) | 30 (8) | 0.7 (2, 98) | 0.482 |
| **Ethnicity, Caucasian %** | 56 (78) | 16 (64) | 3 (100) | 3.8 (2) | 0.151 |
| **Treatment time, months** | 3 (3) | 4 (3) | 1 (0) | 6.6 (2, 98) | <0.001*^b,c^ |

**Supplementary Table 1.** The table compares clinical characteristics across the three studies. Parametric variables were compared using one-way ANOVA and reported as mean (standard deviation, SD) and F statistic (df; degrees of freedom between groups, within groups). Results for Phillips 2013 were reported as median (minimum - maximum). Categorical variables were compared across the three groups using Chi-Square tests and reported as count (percentage, %), and Chi-square test static, χ^2^ (df). Significance denoted, * p<0.005.  ^b A significant difference between Jugé, 2021 and Phillips, 2013
c A significant difference between Jugé, 2021 and Sutherland, 2018
d A significant difference between Sutherland 2018 and Phillips, 2013^
