# Supplementary material for: Effect of mandibular advancement splint therapy on cardiac autonomic function in obstructive sleep apnoea
Source: Sleep Breath. 2023 Sep 28;28(1):349–57. doi: 10.1007/s11325-023-02924-y (PMC10955011; doi:10.1007/s11325-023-02924-y)
Supplement: Supplementary file 3 — Supplementary file3 (DOCX 15 KB) [file 11325_2023_2924_MOESM3_ESM.docx]

| **Change in HRV** | **Sutherland 2018 n = 73** | **Jugé 2021 n = 25** | **Phillips 2013 n = 3** | **Test Statistic** | **p** |
| --- | --- | --- | --- | --- | --- |
| **avgNN _ms_** | 9 (131) | 21 (121) | 71 (25-164) | 2.2  ^a^ | 0.325 |
| **SDNN _ms_** | -8 (13) | -3 (13) | 18 (-1–268) | 3.8  ^a^ | 0.146 |
| **RMSSD _ms_** | -4 (14) | -1 (14) | 9 (2-406) | 4.5  ^a^ | 0.106 |
| **pNN50 ^%^** | -1 (11) | -1 (9) | 5 (1-22) | 3.4  ^a^ | 0.181 |
| **TP _ms_^2^** | -258 (1502) | -297 (1151) | 1585 (-288-142219) | 2.9  ^a^ | 0.234 |
| **LF _ms_^2^** | -51 (458) | -56 (233) | 599 (-39-36668) | 3.4  ^a^ | 0.179 |
| **HF _ms_^2^** | -90 (432) | -43 (357) | 150 (38 -86273) | 4.3  ^a^ | 0.118 |
| **LF: HF** | -1 (1) | -1 (1) | -1 (-1-1) | 1.8  ^a^ | 0.399 |
| **LF_nu_** | 4 (16) | 3 (11) | -6 (23) | 0.6 (2, 98) | 0.545 |
| **HF_nu_** | -5 (15) | -1 (7) | 10 (22) | 2.1 (2, 98) | 0.124 |

**Supplementary Table 2.** The table compares change in HRV markers across the three studies. Nonparametric variables were compared using Kruskal-Wallis Test, denoted ^‘a’^, and reported as median (interquartile range, IQR ) with the H test statistic. Parametric variables were compared using one-way ANOVA and reported as mean (standard deviation, SD) and F statistic (df; degrees of freedom between groups, within groups). Results for Phillips 2013 were reported as median (minimum - maximum). Significance denoted, * p<0.005
