# Supplementary material for: Effect of mandibular advancement splint therapy on cardiac autonomic function in obstructive sleep apnoea
Source: Sleep Breath. 2023 Sep 28;28(1):349–57. doi: 10.1007/s11325-023-02924-y (PMC10955011; doi:10.1007/s11325-023-02924-y)
Supplement: Supplementary file 4 — Supplementary file4 (DOCX 14 KB) [file 11325_2023_2924_MOESM4_ESM.docx]

|  | **Complete Responder**  **N= 36** | **Partial Responder**  **N = 38** | **Non-Responder**  **N= 27** | **Test Statistic** | **p** |
| --- | --- | --- | --- | --- | --- |
| **Age, years** | 53 (12) | 56 (10) | 57 (13) | 0.8 (2, 98) | 0.461 |
| **Sex, male %** | 19 (53) | 18 (47) | 17 (62) | 1.1 (2) | 0.568 |
| **BMI, kg/m²** | 27 (4) | 31 (6) | 30 (5) | 4.7 (2, 98) | 0.011 |
| **Ethnicity, Caucasian %** | 26 (72) | 28 (73) | 18 (67) | 11.4 (2) | 0.524 |
| **Treatment time, months** | 3 (2) | 4 (2) | 4 (2) | 1.1 (2, 98) | 0.450 |

**Supplementary Table 3.** The table compares clinical characteristics across the three response groups. Parametric variables were compared using one-way ANOVA and reported as mean (standard deviation, SD) and F statistic (df; degrees of freedom between groups, within groups). Categorical variables were compared across the three groups using Chi-Square tests and reported as count (percentage, %), and Chi-square test static, χ^2^ (df). Significance denoted, * p<0.005
