# Supplementary material for: Effect of mandibular advancement splint therapy on cardiac autonomic function in obstructive sleep apnoea
Source: Sleep Breath. 2023 Sep 28;28(1):349–57. doi: 10.1007/s11325-023-02924-y (PMC10955011; doi:10.1007/s11325-023-02924-y)
Supplement: Supplementary file 5 — Supplementary file5 (DOCX 15 KB) [file 11325_2023_2924_MOESM5_ESM.docx]

| **Change in HRV** | **Complete Responder**  **n= 36** | **Partial Responder**  **n = 38** | **Non-Responder**  **n= 27** | **Test Statistic** | **p** |
| --- | --- | --- | --- | --- | --- |
| **avgNN _ms_** | 45 (173) | 13 (114) | 5 (120) | 2.9  ^a^ | 0.234 |
| **SDNN _ms_** | -3 (18) | -4 (15) | -7 (10) | 1.2  ^a^ | 0.532 |
| **RMSSD _ms_** | -2 (25) | -2 (11) | -3 (12) | 0.3  ^a^ | 0.831 |
| **pNN50 ^%^** | -1 (16) | -1 (8) | -3 (11) | 1.4  ^a^ | 0.492 |
| **TP _ms_^2^** | -259 (2227) | -95 (1282) | -499 (982) | 1.2  ^a^ | 0.540 |
| **LF _ms_^2^** | -66 (421) | -3 (376) | -88 (334) | 2.0  ^a^ | 0.362 |
| **HF _ms_^2^** | -22 (1067) | -50 (316) | -137 (333) | 0.9  ^a^ | 0.909 |
| **LF: HF** | 1 (1) | 1 (1) | -1 (1) | 3.1  ^a^ | 0.210 |
| **LF_nu_** | 3 (15) | 4 (13) | 1 (15) | 0.6 (2, 98) | 0.540 |
| **HF_nu_** | -5 (14) | -5 (15) | -4 (12) | 0.5 (2, 98) | 0.612 |

**Supplementary Table 4.** The table compares change in HRV markers across the three response subgroups. Nonparametric variables were compared using Kruskal-Wallis Test, denoted ^‘a’^, and reported as median (interquartile range, IQR ) with the H test statistic. Parametric variables were compared using one-way ANOVA and reported as mean (standard deviation, SD) and F statistic (df; degrees of freedom between groups, within groups). Significance denoted, * p<0.005
Complete responders (n = 36), defined as patients with > 50% improvement in AHI as well as a posttreatment AHI < 5/h; Partial responders (n =38), defined as patients with > 50% AHI reduction but still had residual OSA (AHI > 5/h), Non- Responders (n= 27), defined as patients with < 50% AHI reduction.
