# Supplementary material for: Effect of mandibular advancement splint therapy on cardiac autonomic function in obstructive sleep apnoea
Source: Sleep Breath. 2023 Sep 28;28(1):349–57. doi: 10.1007/s11325-023-02924-y (PMC10955011; doi:10.1007/s11325-023-02924-y)
Supplement: Supplementary file 6 — Supplementary file6 (DOCX 15 KB) [file 11325_2023_2924_MOESM6_ESM.docx]

| **Change in HRV** | **1-4 months**  **n= 74** | **5-8 months n = 21** | **9-12 months n= 6** | **Test Statistic** | **p** |
| --- | --- | --- | --- | --- | --- |
| **avgNN _ms_** | 14 (128) | 5 (125) | 90 (131) | 5.1  ^a^ | 0.078 |
| **SDNN _ms_** | -4 (13) | -2 (15) | 5 (14) | 2.7  ^a^ | 0.257 |
| **RMSSD _ms_** | -3 (15) | -2 (18) | -2 (22) | 1.6  ^a^ | 0.449 |
| **pNN50 ^%^** | -1 (11) | -1 (14) | -1 (24) | 1.9  ^a^ | 0.392 |
| **TP _ms_^2^** | -309 (1462) | -120 (1555) | 403 (1330) | 3.2  ^a^ | 0.200 |
| **LF _ms_^2^** | -68 (360) | -51 (343) | 127 (385) | 2.9  ^a^ | 0.235 |
| **HF _ms_^2^** | -92 (400) | -46 (530) | -32 (637) | 1.8  ^a^ | 0.399 |
| **LF: HF** | 1 (1) | 1 (1) | 1 (3) | 0.2  ^a^ | 0.917 |
| **LF_nu_** | 3 (16) | 3 (14) | 11 (18) | 0.7 (2, 98) | 0.488 |
| **HF_nu_** | -3 (14) | -4 (16) | -3 (14) | 0.1 (2, 98) | 0.866 |

**Supplementary Table 5.** The table compares change in HRV markers across the three treatment time groups. Nonparametric variables were compared using Kruskal-Wallis Test, denoted ^‘a’^, and reported as median (interquartile range, IQR ) with the H test statistic. Parametric variables were compared using one-way ANOVA and reported as mean (standard deviation, SD) and F statistic (df; degrees of freedom between groups, within groups). Significance denoted, * p<0.005
